# Genetic Diversity in the Lesser Antilles and Its Implications for the Settlement of the Caribbean Basin

**DOI:** 10.1371/journal.pone.0139192

**Published:** 2015-10-08

**Authors:** Jada Benn Torres, Miguel G. Vilar, Gabriel A. Torres, Jill B. Gaieski, Ricardo Bharath Hernandez, Zoila E. Browne, Marlon Stevenson, Wendell Walters, Theodore G. Schurr, The Genographic Consortium

**Affiliations:** 1 Department of Anthropology, University of Notre Dame, Notre Dame, Indiana, United States of America; 2 Department of Anthropology, University of Pennsylvania, Philadelphia, Pennsylvania, United States of America; 3 Missions Programs, National Geographic Society, Washington, D.C., United States of America; 4 Santa Rosa First Peoples Community, Arima, Trinidad and Tobago; 5 The Garifuna Heritage Foundation Inc., Kingston, St. Vincent and the Grenadines; 6 Sandy Bay Village, St. Vincent and the Grenadines; Universitat Pompeu Fabra, SPAIN

## Abstract

Historical discourses about the Caribbean often chronicle West African and European influence to the general neglect of indigenous people’s contributions to the contemporary region. Consequently, demographic histories of Caribbean people prior to and after European contact are not well understood. Although archeological evidence suggests that the Lesser Antilles were populated in a series of northward and eastern migratory waves, many questions remain regarding the relationship of the Caribbean migrants to other indigenous people of South and Central America and changes to the demography of indigenous communities post-European contact. To explore these issues, we analyzed mitochondrial DNA and Y-chromosome diversity in 12 unrelated individuals from the First Peoples Community in Arima, Trinidad, and 43 unrelated Garifuna individuals residing in St. Vincent. In this community-sanctioned research, we detected maternal indigenous ancestry in 42% of the participants, with the remainder having haplotypes indicative of African and South Asian maternal ancestry. Analysis of Y-chromosome variation revealed paternal indigenous American ancestry indicated by the presence of haplogroup Q-M3 in 28% of the male participants from both communities, with the remainder possessing either African or European haplogroups. This finding is the first report of indigenous American paternal ancestry among indigenous populations in this region of the Caribbean. Overall, this study illustrates the role of the region’s first peoples in shaping the genetic diversity seen in contemporary Caribbean populations.

## Introduction

**Fig 1:**
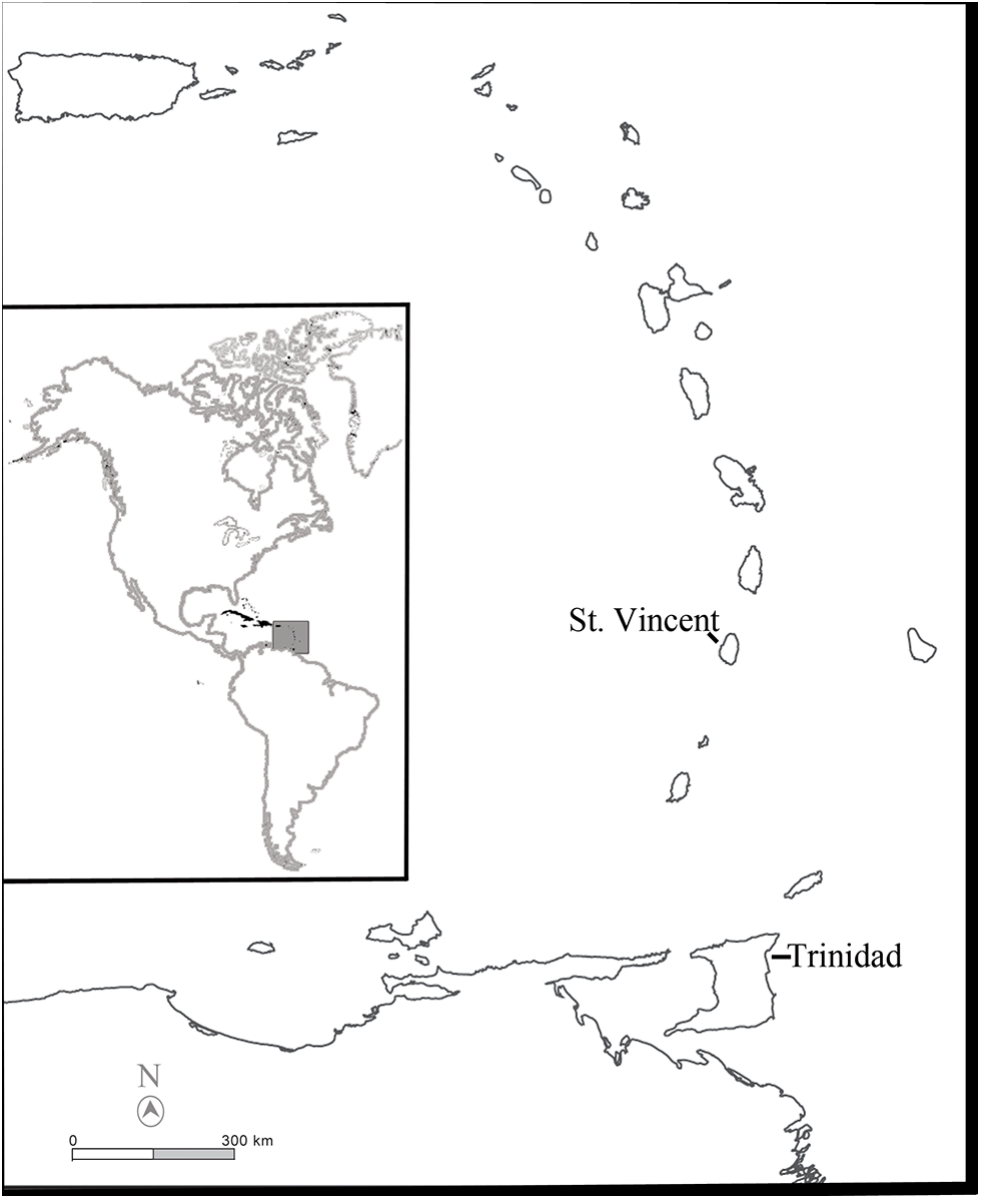
A map of the Lesser Antilles showing the locations of St. Vincent and the Grenadines and Trinidad and Tobago.

The Caribbean is a vast region encompassing nearly 3 million km^2^ in total area. Bordered by the Atlantic Ocean to in the east, it spans from the southern coast of the Bahamas to the northern coast of South America and the eastern coast of Mexico and Central America (**Fig 1**). Comprised of well over 700 islands, this region is currently home to an estimated 17 million people [1].

The initial presence of human populations within the region has been dated to 7,200 years before present (ybp) based on evidence from the Banwari Trace site in Trinidad [2]. Aside from this site, the first human settlement in the Caribbean dates to a migration event around 8,000–5,000 ybp marked by sites on Cuba, Hispañola and Puerto Rico [3–5]. Within the northern Lesser Antilles and Barbados, human occupation dates back as far as 5,000–3,000 ybp [3]. Both archeological and linguistic research suggest that, for the remaining islands of the Antilles, a series of migrations ending just prior to 1500 AD resulted in human presence throughout the region [6]. Possible sources of these population expansions to the southern Lesser Antilles include northern South America or, alternatively, the Greater Antilles through southward migrations [6–15].

As a result of these different expansions, at least eight different ethnic groups were present in the Caribbean at the time of European contact. These include the Guanahatebay in Cuba, the Macorix of Hispañola, the Ciguayo of Hispañola, the Lucayo (also referred to as Lucayan Taíno) in the Bahamas, the Ciboney of Haiti, Jamaica, and Cuba, the Classic Taíno in the Dominican Republic, Puerto Rico, the Virgin Islands, and the Leeward Islands, and the Kalipuna and the Karina Caribs in Windward islands of the Lesser Antilles [7,16]. The exact cultural, biological and linguistic affinities of these historical populations have yet to be fully determined.

Indigenous Caribbean peoples were greatly affected by the assimilation, disease, and genocide brought about by European colonization and the Trans-Atlantic slave trade [17]. These communities were also systematically dispersed to other islands or parts of the Americas during the colonial period, as well as legislated or otherwise written out of the historiography of the region [18–22]. Despite the colonial era population decline [15,23], several contemporary indigenous communities still persist on different islands of the Caribbean.

While archeological, ethnohistorical, and linguistic data provide crucial perspectives on the peopling and history of the Caribbean Basin, many questions regarding the timing and origins of the initial migrations and the impact of European colonization on indigenous Caribbean communities remain unanswered. In response to these issues, and with local and community consent, we characterized mitochondrial DNA and Y-chromosome variation in two indigenous communities, the Santa Rosa First Peoples’ Community (FPC) in Trinidad and the Garifuna of St. Vincent. The resulting data allowed us to examine the plausibility of different models regarding the purported origins of the initial migrations into the Caribbean Basin and evaluate hypotheses about the genetic relationships between indigenous Caribbean and circum-Caribbean populations. These data were also used to assess how the entry of African, Asian and European peoples into the Caribbean has affected the demography of indigenous populations of the Lesser Antilles.

## Methods

### Ethics statement

Prior to study commencement, this project was reviewed and approved by the University of Pennsylvania IRB #8 and the University of Notre Dame IRB. In addition, prior to sample collection, we obtained approval from the National Ethics Research Committee, Ministry of Health, Wellness and the Environment, St. Vincent and the Grenadines, and the Ethics Committee, Government of the Republic of Trinidad and Tobago, and the Santa Rosa FPC and St. Vincent Garifuna Community. Each participant also provided written informed consent prior to sample collection and documentation of family history information.

### Indigenous Caribbean communities

The Santa Rosa FPC is based in Arima, a settlement located in the north-central region of Trinidad, roughly 26 km east of Port of Spain. According to the Trinidadian Central Statistics Office 2011 census, approximately 1,328 people in Trinidad identified as indigenous, 7% of whom live in Arima [24]. The history of the FPC extends back to when Trinidad was a Spanish colony [25]. In 1749, the Indian Mission of Arima was established in the north-central region of Trinidad as a Capuchin Mission town [25,26]. This mission town was named “Arima” in honor of Hyarima, an indigenous leader and activist who during the 16^th^ century led several rebellions to defend his community from colonial encroachment [27]. As part of a colonial initiative to annex native lands, all indigenous people in the remaining Trinidadian missions towns were consolidated into the Santa Rosa Mission [22]. Although this mission was formally dissolved in 1849, many indigenous peoples remained in the vicinity and the Spanish influence on the indigenous community remains evident as many contemporary indigenous peoples have Spanish surnames [21]. In 1976, the Santa Rosa FPC was incorporated as a limited liability company, thereby becoming the only collective of indigenous people that have national recognition within Trinidad [22,25,28].

Like that of their counterparts in Trinidad, the history of the Garifuna of St. Vincent is a chronicle of resistance and survival [20]. The Garifuna are the descendants of indigenous Caribbean peoples who fiercely resisted British colonization into the 18^th^ century. During this period, France and Britain agreed to leave St. Vincent, Dominica, St. Lucia, and Tobago as so-called ‘neutral islands’ for the indigenous people of the Caribbean. In reality, while both France and Britain worked to ensure that the other would gain no advantage on the neutral islands, Britain formally seized control of St. Vincent in 1763. British encroachment on indigenous lands resulted in the First Carib War (1772–73), after which the British succeeded in dispossessing indigenous peoples and forcing them into a treaty. Although the Vincentian indigenous groups halted the British military advance at that time, within twenty years, the Second Carib War (1795–96) erupted between these antagonists. As a result of this conflict, Vincentian indigenous peoples were deported, first to the nearby island of Balliceaux, and then to Roatán, an island located off of the coast of Honduras [20,29].

Prior to its becoming a colony, both the British and the French employed ethnic distinctions to divide indigenous Vincentian peoples and help them annex native lands [30]. Accordingly, those thought to be descendants of indigenous Caribbean and African ancestors were called ‘Black Caribs’, while those thought to be descendants of only the original inhabitants of the region were called ‘Yellow Caribs’. The ‘Black Caribs’ were forcibly removed from St. Vincent and relocated to different parts of Central America, while the ‘Yellow Caribs’, although initially exiled, were returned to the island [20]. As a consequence of this forced migration, more than half of the exiled Vincentian population was lost, although some survivors eventually returned to St. Vincent. Today, contemporary Garifuna peoples are found in countries throughout Central America, on St. Vincent, and in the United States [31,32]. The native communities of St. Vincent are generally located in the north of the island at Sandy Bay, Fancy, and Owia, but also in the southwest regions at Grieggs Point, Rose Bank, and Rose Hall [33].

### Sampling

Genetic samples, collected via buccal swabs, were obtained from a total of 88 indigenous individuals in both Trinidad and St. Vincent. Of this total, 65 participants were Garifuna from the Kingstown area and two regions on the northeastern coast of St. Vincent. The remaining 23 samples were obtained from members of the Santa Rosa FPC in Arima, Trinidad. All 88 DNA samples were subjected to genetic analysis. After review of the genealogical data, we removed maternal or paternal relatives from the sample set, leaving 7 Santa Rosa FPC and 25 Garifuna females and 5 Santa Rosa and 18 Garifuna male samples for statistical and phylogenetic analyses. Although these sample sizes are small for standard statistical analyses, both communities are small, demographically speaking, numbering under 2,000 people, including relatives. Consequently, the small study sample sizes reflect the communities from which they were derived. While these sample sizes may affect how robust the statistical and phylogenetic analyses described below, they nonetheless allow for some generalizations about human prehistory in this region of the Caribbean.

### Mitochondrial DNA and Y-chromosome analysis

The mitochondrial DNAs (mtDNAs) from these samples were analyzed by using direct sequencing and single nucleotide polymorphism (SNP) genotyping methods as previously described, with the entire control region (CR) (np 16024–576) being sequenced in each sample [34–36]. The resulting mtDNA sequences were aligned, edited, and compared to the rCRS [37] in Sequencher, v 4.9 [38]. Haplogrep [39], an automated online web application based on Phylotree15 [40], was used to identify mtDNA haplogroups based on hypervariable segment 1 and 2 (HVSI and HVSII) polymorphisms. The coding region SNPs and CR sequences defined the maternal haplogroup and haplotypes, respectively, for each individual. Following this basic sequence characterization, haplogroup frequencies were calculated by hand and summary statistics such as nucleotide and haplotype (gene) diversity were estimated from mtDNA sequence data using DNASp [41].

We determined the paternal genetic ancestry of the St. Vincent and Trinidadian male participants by screening the non-recombining region of the Y-chromosome (NRY) for 17 phylogenetically informative biallelic markers (SNPs) that define paternal haplogroups and their major sub-branches (L54, M3, M9, M19, M45, M89, M96, M168, M173, M194, M199, M207, M242, M253, M343, P215, SA01) [42–44]. All markers were screened using custom TaqMan assays, and scored on an ABI 7900HT Fast Real-Time PCR System [34–36].

Paternal haplotypes were further defined through the analysis of 17 Y-chromosome short tandem repeats (Y-STRs) that are part of the AmpFℓSTR Y-filer Amplification Kit (ABI). A separate custom multiplex reaction was also used to characterize six additional SNPs (M17, M60, M91, M139, M175, and M186) and two additional Y-STRs (DYS388, and DYS426). PCR products were run with GeneScan 500 LIZ Size Standards and read on an ABI 3130xl Gene Analyzer [34–36,43].

We used Haplogroup Predictor [45]to assign all Y-STR haplotypes to paternal haplogroups. Due to the possible convergence of Y-STR allele sizes in different NRY haplotypes [46,47], we confirmed the haplogroup predictions by genotyping the relevant diagnostic SNPs using custom TaqMan assays (see above). The Y-chromosome haplogroup frequencies were calculated by hand, while summary statistics were estimated using Arlequin v.3.11 software [48].

When comparing the St. Vincent and Trinidad communities to other Caribbean populations, we used data from the Y-STR loci recommended by the Scientific Working Group on DNA Analysis Methods (SWGDAM) [49]. This set included the DYS19, DYS385a, DYS385b, DYS389I, DYS389II, DYS390, DYS391, DYS392, DYS393, DYS438, and DYS439 loci. In these analyses, DYS385 data from published studies were used in the diversity estimates. Here, the shorter repeat allele was consistently associated with DYS385a, although the assignment of the two-repeat alleles cannot be accurately made without further genotyping.

#### Comparative analyses

To assess the population affinities of the St. Vincent and Trinidadian populations, we compared their mtDNA HVS1 sequences (np 16024–16400) to those of several North American [50], Caribbean [51,52], and circum-Caribbean populations in Central and South America [50,53–58]that were available in Genbank (Table 1). Comparative analyses included estimates of diversity and exact tests for population differentiation. F_ST_, a measure of population differentiation due to genetic structure, was estimated based on HVS1 sequences for Vincentian, Trinidadian and comparative populations with Arlequin v.3.11 [48] using the Tamura-Nei distance method with a gamma correction of 0.47 [59]. The inter-population F_ST_ estimates were subsequently visualized through multi-dimensional scaling (MDS) [60]using SPSS 20.0. The geographic location of each comparative population obtained from the literature, and the corresponding language family ascertained from Ethnologue [61], were then incorporated into the MDS plot.

**Table 1:** Comparative mitochondrial DNA sequences used in the current study.

Geographic region		Source
Amazonian Basin	Brazil	1, 2, 3
Northern Tropics	Venezuela	4, 5
	Colombia	2, 6
Mesoamerica	Panama	2, 6, 7
	Costa Rica	3
	Guatemala	3
Caribbean	Puerto Rico	8
	Anglophone Islands*	9

Sources: 1 = Fagundes et al., 2008; 2 = Perego et al. 2010; 3 = Yang et al., 2010; 4 = Castro de Guerra et al., 2012; 5 = Vona et al., 2005; 6 = Tamm et al., 2007; 7 = Perego et al., 2012; 8 = Vilar et al., 2014; 9 = Benn Torres et al., 2007

In addition, we constructed median-joining (MJ) networks with MP post-processing function [62]in Network 4.6.1.0 [63,64]to analyze the phylogenetic relationships among HVS1 sequences from the current study and comparative populations. Two MJ networks were constructed, the first for sequences belonging to haplogroup A2 and the second for those belonging to haplogroup C1. Because of their shorter lengths and general lack of Native American mitochondrial haplogroups, the 314 comparative Anglophone Caribbean sequences [51], listed in Table 1, were not used in the network analyses. The coalescence time for each network was calculated using the HVSI mutation rate of 1.64273 x 10^−7^ per nucleotide per year [65].

The Y-STR data from St. Vincent and Trinidad were compared to those from published studies of Native American populations [66–70](Table 2). In this comparative analysis, only data from indigenous American Y-chromosomes were used. After adjusting the data sets to include only SWGDAM loci, we calculated summary statistics including gene diversity, average gene diversity and shared haplotypes, and estimated genetic distances between populations, R_ST,_ using Arlequin v.3.11 software [48]. The R_ST_ estimates were then visualized in a multidimensional scaling (MDS) plot in SPSS [71]. In addition, genetic differentiation between the indigenous Caribbean populations (Trinidadian and Vincentian samples were combined into one group) and the comparative populations was tested using exact tests in Arlequin v.3.11 [48].

**Table 2:** Comparative Y-STR data used in the current study.

Geographic region		Source
Amazonian Basin	Brazil	1
Northern Tropics	Venezuela	2 [66]
	Colombia	3
	French Guiana	4 [70]
Central America	Honduras	5 [69]

Sources: 1 = Leite et al., 2008; 2 = Borjas et al., 2008; 3 = Romero et al., 2008; 4 = Mazieres et al., 2010; 5 = Matamoros et al., 2009

MJ network and coalescence analyses [63,64]were also used to explore the phylogenetic relationships among Y-chromosomes from indigenous Caribbean and South American populations. In the MJ network analysis, each Y-STR locus was weighed according to its mutation rate as listed in STRBase [72], with slower rates given more weight than those with faster rates. In the coalescence analysis, we used an evolutionary mutation rate (EMR) of 2.8 x10^-5^ mutations per locus per year [73], one shown by Wei and colleagues [74]to produce time to most recent common ancestor (TMRCA) values that most closely corresponded with estimates generated from resequencing data.

## Results

### mtDNA diversity in St. Vincent and Trinidad

For the two populations, we detected maternal indigenous ancestry in 42% of the individuals (Table 3; Table A in S1 File). However, only two of the five major founding Native American mitochondrial haplogroups, A2 and C1, were detected in these samples, with haplogroups B2, D1, and X2a being absent. The distribution of these two haplogroups varied between the islands, with haplogroup A2 representing 42% and haplogroup C1 17% of the total mtDNAs in the Trinidadian population, and A2 comprising only 16% and C1 21% of mtDNAs in St. Vincent, respectively.

**Table 3:** Mitochondrial DNA and Y-chromosome haplogroup frequencies in indigenous Caribbean communities.

mtDNA Haplogroup	Trinidad % (n)	St. Vincent % (n)	NRY Haplogroup	Trinidad % (n)	St. Vincent % (n)
A2	41.7 (5)	16.3 (7)	E1b1a	60(3)	44.4(8)
C1	16.7 (2)	20.9 (9)	Q-M3	20(1)	16.7(3)
L0	0 (0)	7 (3)	R1b	20(1)	22.2(4)
L1	0 (0)	4.7 (2)	I1	-	11.1(2)
L2	16.7 (2)	30.2 (13)	I2b	-	5.5(1)
L3	16.7 (2)	20.9 (9)			
M33	8.3 (1)	-			

In addition to indigenous Caribbean ancestry, both communities exhibited maternal lineages from Africa and, in the case of the Trinidadians, South Asia. Most of the non-indigenous lineages in both communities belonged to African haplogroups L0, L1, L2, and L3. Haplogroup L2 was the most common African lineage among the Vincentians, while L2 and L3 were the most common lineages in FPC Trinidadians. In addition, FPC Trinidadians had mtDNAs belonging to haplogroup M33a, a lineage commonly seen in northeastern India [75].

Analysis of mtDNA CR sequences revealed a total of 58 distinct haplotypes in the St. Vincent Garifuna and the FPC Trinidadians. Of these, 23 belonged to indigenous American lineages, 31 to African lineages, and 2 to South Asian lineages (Table B in S1 File). When the CR sequences were reduced to the HVS1 sequence (np 16024–16390), we observed 8 Native American, 24 African, 1 South Asian haplotypes (Table C in S1 File). These HVSI sequences have been deposited to Genbank, accession numbers KT777741-KT777798 (Table D in S1 File **).** Furthermore, these HVS1 sequences were used for all subsequent statistical and phylogenetic analyses.

As indicated by the summary statistics based on HVS1 sequences, the FPC Trinidadians exhibited higher gene diversity than the Vincentian Garifuna. The indigenous Caribbean communities also had generally similar levels of genetic diversity relative to Puerto Ricans and most other comparative populations (Table**s** 4 and 5). However, the indigenous Caribbean communities also exhibited slightly lower diversity estimates compared to other Caribbean populations within the Lesser Antilles, excepting the Dominicans (Dominica). Unlike the other Anglophone island samples, those of the Dominicans were obtained from a rural population, with some of its members having genealogical ancestry linking them to the indigenous people of Dominica [51]. The observed differences could be a function of the small sample size for both the FPC Trinidadian and Vincentian Garifuna, which are small communities numbering fewer than 2000 people each [24,76].

**Table 4:** MtDNA summary statistics for the Indigenous Caribbean communities based on HVS1 sequences (np 16024–16400).

HVS1 (np 16024–16400)	n	Haplotypes	H (± SD)	π (± SD)
All mtDNA lineages				
St. Vincent	43	25	0.942 (0.021)	0.020 (0.002)
Trinidad	12	10	0.955 (0.057)	0.018 (0.002)
Native American mtDNA lineages only				
St. Vincent	16	4	0.650 (0.075)	0.012 (0.001)
Trinidad	7	5	0.857 (0.004)	0.013 (0.004)

**Table 5:** Summary statistics for mtDNA HVS1 sequences (np 16024–16400) from comparative populations.

Geographic region		n	# Haplotypes	H (±SD)	π (±SD)
Amazonian Basin	Brazil	62	32	0.956 (0.013)	0.053 (0.029)
Northern Tropics	Venezuela	163	99	0.964 (0.005)	0.063 (0.033)
	Colombia	129			
Mesoamerica	Panama	40	28	0.792 (0.050)	0.036 (0.021)
	Costa Rica	15			
	Guatemala	16			
Caribbean	Puerto Rico	186	24	0.866 (0.013)	0.017 (0.009)
	Anglophone Islands*	314	183	0.991 (0.001)	0.029 (0.001)

^*^Only sequences from np 16109–16393 were used in the comparison

In the exact test of population differentiation, the two indigenous Caribbean populations were significantly different from one another as well as from several comparative populations from Brazil, Colombia, Costa Rica, and Panama (Table E in S1 File). The Garifuna tended to be more diverged from South American groups than the Trinidad FPC. Comparisons of estimates of genetic diversity also indicated that the indigenous Caribbean communities had a pattern of genetic variation distinct from those of the surrounding communities from the same island. These patterns could change with the addition of more Santa Rosa FPC and Vincentian Garifuna samples.

**Fig 2:**
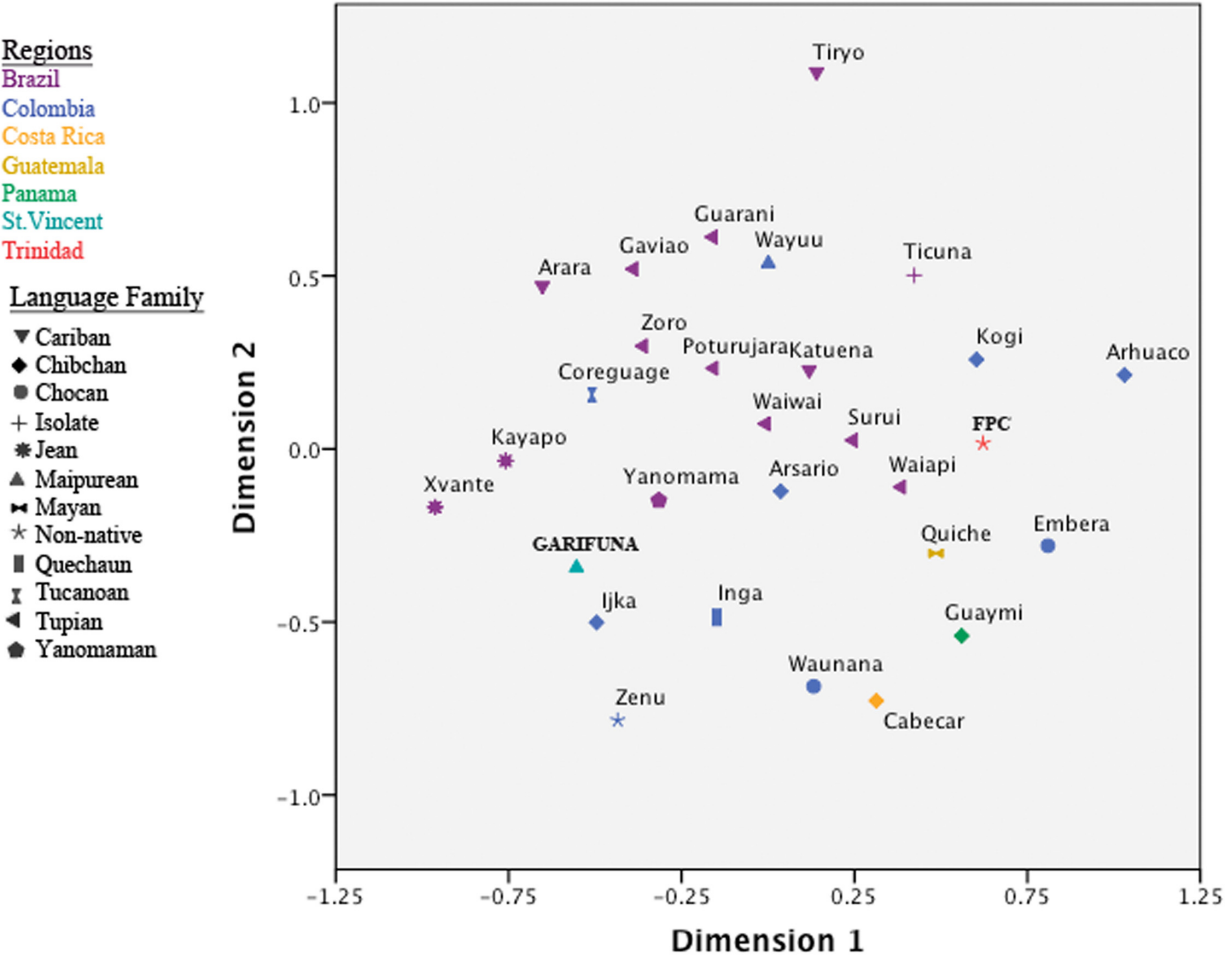
A MDS plot of FST estimates based on mtDNA HVS1 sequences (np 16024–16400) for Indigenous Caribbean and comparative Central and South American populations. The stress value of the plot is 9.8%. Data points are labeled with the population name and color-coded by geographic origin, with each shape corresponds to the language family of the sample.

MDS was used to visualize *F*_ST_ estimates between the indigenous Caribbean and comparative populations from North America, the Caribbean, Central America, and South America (Fig 2; Table F in S1 File). For this analysis, only indigenous American haplotypes were used. To investigate the relationship between geography and language, comparative urban populations without an identified ethnicity or language affiliation were removed from the analysis. In addition, in the initial analyses, we included data from comparative North American populations. However, their inclusion did not clarify the population affinities for the Caribbean groups (data not shown) and, as a result, the North American groups were excluded from the final comparative analysis.

Although these results may have been influenced by the small sample sizes for the indigenous Caribbean communities, the Vincentian Garifuna and FPC Trinidadian populations were not separated from South and Central American populations, falling near the central region of the MDS plot. Interestingly, the indigenous Caribbean populations were somewhat distant from each other, with several South American populations interspersed between them. In this regard, the FPC Trinidadians appeared closer to some Brazilian, Colombian, and Central American populations, whereas the Vincentian Garifuna showed greater affinities with Colombian and a different set of Brazilian populations (Fig 2). However, the indigenous Caribbean populations did not fall into any specific language clusters within the MDS plot. While Jean (Gê) speakers formed a cluster along Dimension 1, and Tupian speakers were concentrated in the center of the plot along Dimension 2, both the Vincentian Garifuna and the FPC Trinidadians were positioned outside of them.

**Fig 3:**
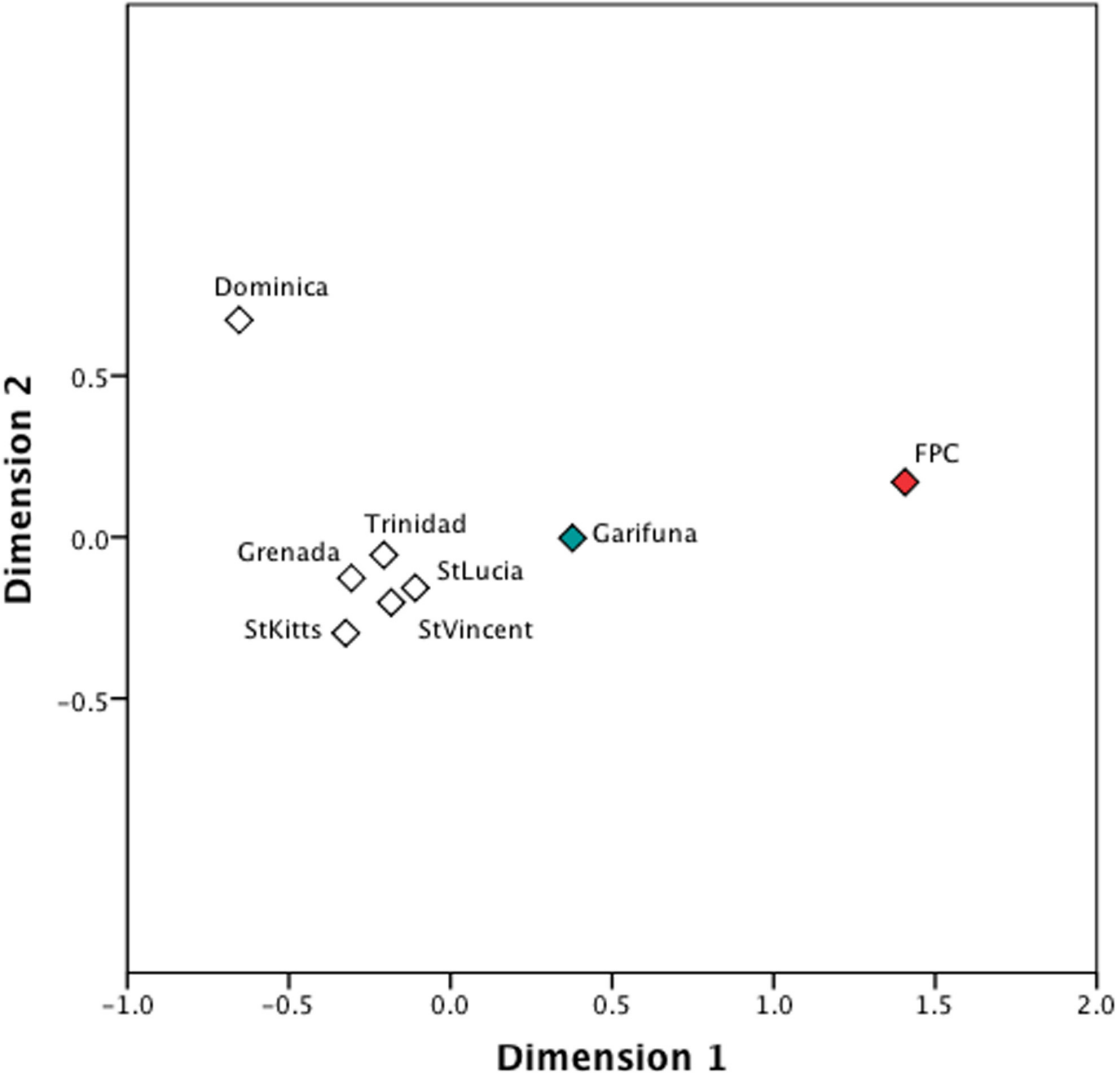
A MDS plot of FST estimates based on mtDNA HVS1 sequences (np 16109–16393) for Indigenous Caribbean and comparative Anglophone Caribbean populations. The stress value of the plot is 0.2%.

We also examined the genetic relationships between the indigenous Caribbean groups and the greater populace in Anglophone Caribbean islands. In this analysis, all haplotypes regardless of their haplogroup identity were used for the *F*_ST_ estimates (Table F in S1 File.). The resulting MDS plot showed most of the islands to be genetically similar to each other (Fig 3). Interestingly, the FPC Trinidadians, the Vincentian Garifuna, and the Dominican populations were genetically distant from the other island populations, as well as separated from each other. While the FPC Trinidadians were genetically distant from most other populations, the Vincentian Garifuna aligned more closely with other Anglophone island populations. The position of the FPC Trinidadians in the plot may reflect its small sample size or perhaps its high frequency of A2 mtDNAs, which are largely absent in the comparative Anglophone island populations. Conversely, the position of the Vincentian Garifuna in the MDS plot may reflect their dual African and indigenous Caribbean ancestry and subsequent cultural and genetic exchange with indigenous Caribbean peoples, as documented by historical sources [20,77].

MJ networks were created from indigenous HVS1 sequences belonging to haplogroups A2 and C1 to determine the phylogenetic relationships between indigenous Caribbean and comparative populations. In the initial analyses involving all of the comparative populations, Mesoamerican groups fell into a separate section of the networks, suggesting a strong geographic structuring of the variation in the Americas (data not shown). Thus, to clarify the genetic affinities of the indigenous Caribbean samples, the Mesoamerica populations were removed from subsequent network analyses.

**Fig 4:**
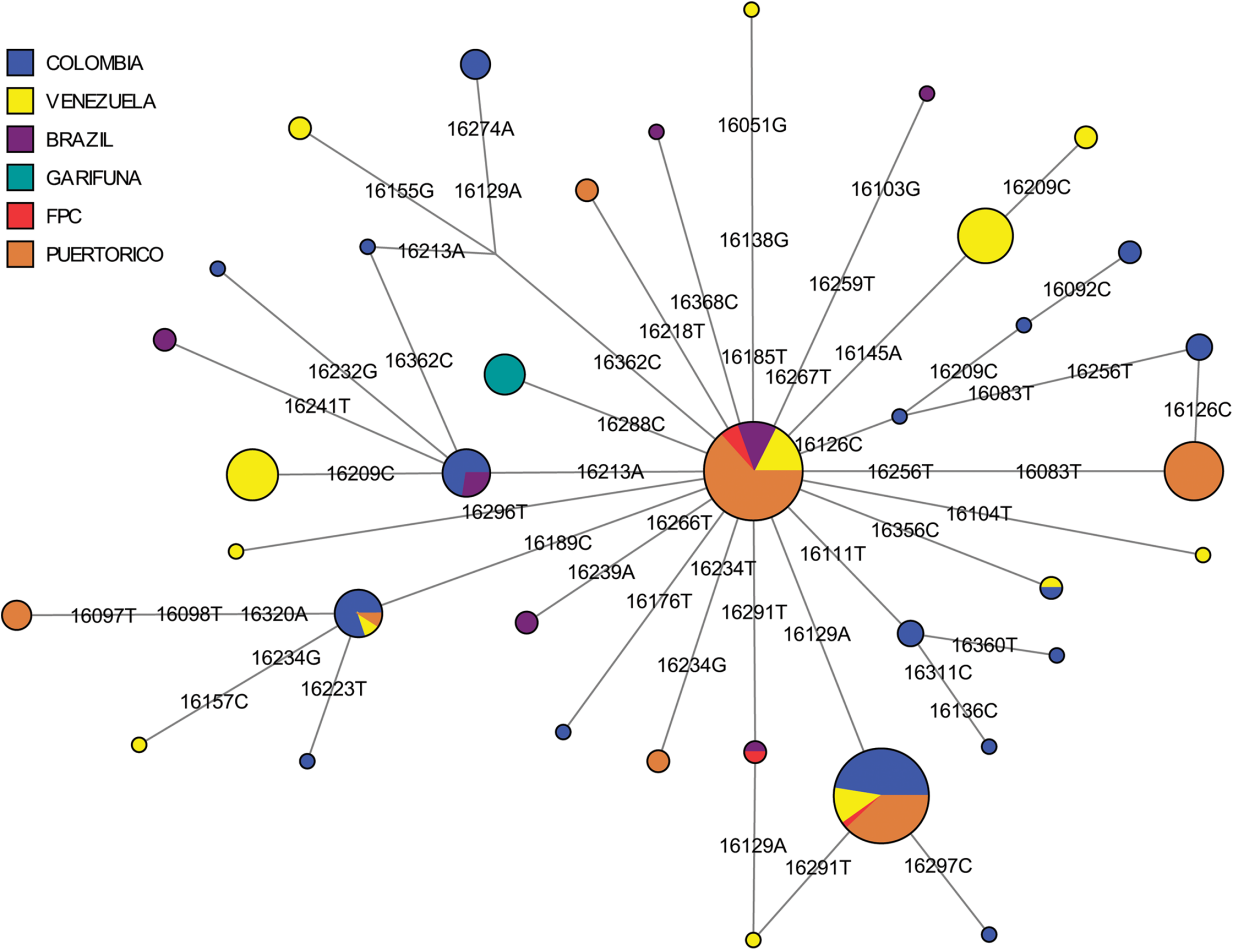
A median-joining network of mtDNA haplogroup A2 haplotypes (np16024-16400) from Indigenous Caribbean and comparative Central and South American populations. Populations represented in each node of the network are shown in different colors.

In the refined A2 MJ network, haplotypes from Trinidad, Puerto Rico, Venezuela, and Brazil comprised the central node of the network (Fig 4). An adjoining haplotype defined by the T16288C mutation was observed only in St. Vincent (#1;Table C in S1 File). This specific haplotype rarely occurs in the Americas, having been seen in only a single Bella Coola individual from the Pacific Northwest [78]. Haplotypes with the T16288C in addition to other polymorphisms have been observed in the Mixe in Mexico [79]and in the Pilaga and Wichi from Argentina [80]. However, unlike the Mixe sequences, those of the Indigenous Caribbean samples had a T195C polymorphism in the HVS2 (Table B in S1 File). Because the mtDNA data from the Bella Coola and Argentinian populations lacked HVS2 data, it was not possible to determine whether the T195C was present in them. These lines of evidence tentatively suggest that the Vincentian haplotype arose through an independent T16288C mutation rather than being genealogically linked to those seen in the Bella Coola or other indigenous populations, although mitogenome sequence data will be required to fully resolve this question.

**Fig 5:**
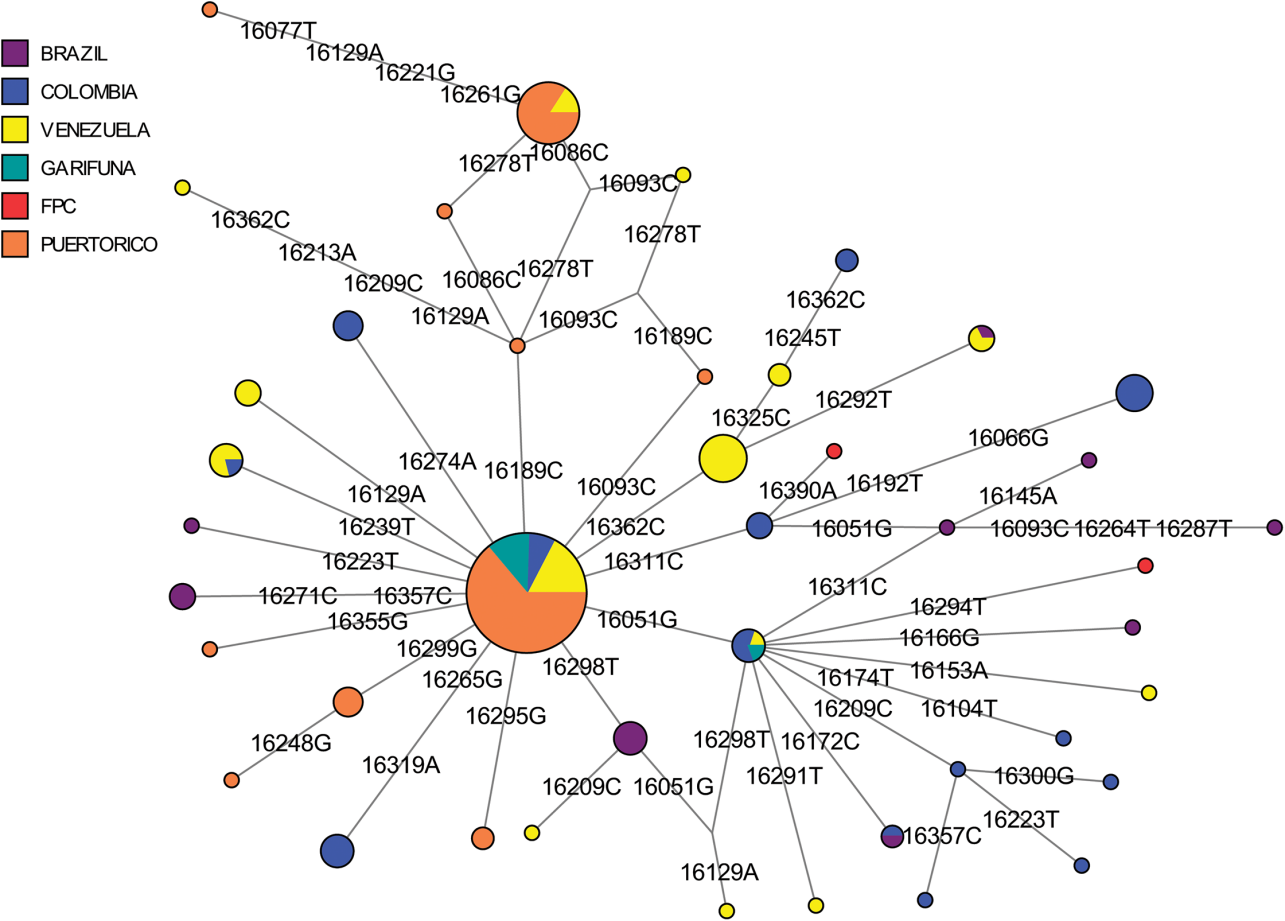
A median-joining network of mtDNA haplogroup C1 haplotypes (np16024-16400) from indigenous Caribbean and comparative Central and South American populations. Populations represented in each node of the network are shown in different colors.

In the refined haplogroup C1 MJ network (Fig 5), Vincentian samples comprised a large proportion of the C1 founder and C1d haplotypes. This was a slightly different pattern than seen in Trinidad and Puerto Rico [56]. While many of the Puerto Rican samples belong to the C1 rather than C1d founder haplotype, one FPC Trinidadian haplotype (#8,Table **C** in S1 File) differed from other C1d haplotypes at C16294T and another FPC Trinidadian haplotype (#6, Table. C in S1 File) was two polymorphisms (T16311C and G16390A) different from the founder C1 haplotype. Both haplotypes #6 and #8 appeared to be unique, as identical haplotypes were not identified among published Genbank sequences, and also lacked the A493G mutation in HVS2 that defines C1b (see #23 and #25; Table **B** in S1 File). In addition, an unusual C1 haplotype occurring in only the SVG Garifuna (#24, Table **B** in S1 File; #7,Table **C** in S1 File) had both the A16051G mutation seen in C1d and the A493G mutation seen in C1b. Although the haplogroup to which it belonged was not entirely clear from the CR sequence, it is likely to be a C1d haplotype with an independent occurrence of the A493G mutation. Overall, in both the A2 and C1 networks, the indigenous Caribbean groups shared only founder haplotypes with other comparative populations, including Puerto Ricans [52].

Coalescence analysis indicated that the indigenous Caribbean A2 haplotypes were older than those from haplogroup C1, with A2 dating to 8489 (± 7095) and C1 to 2452 (± 1501) ybp, respectively. While the age of C1 is generally consistent with estimate based on our mtDNA data from the Greater Antilles, that of A2 was not [55]. The difference in age estimates between the Lesser and Greater Antilles, as well as the large standard errors associated with these dates, may be attributable to the small number of haplotypes included in the estimates, the possible influence of founder effects in reducing haplotype diversity, or the inclusion of multiple distinct sublineages of A2 haplotypes that inflate the coalescence estimate.

### Y-chromosome DNA diversity in St Vincent and Trinidad

African, European, and Native American Y-chromosome haplogroups were present in both the Trinidadian and Vincentian populations (Table 3; Table **G** in S1 File). Within the indigenous Caribbean groups, four individuals had the ubiquitous haplogroup Q1a3a1a1 (Q-M3) haplotypes, while none had any from the less common Q1a3a1 (Q-L54). Haplotypes from haplogroup C3c, another Native American paternal lineage that is common among Na-Dene speakers and is restricted to North America [81], were not observed in the Caribbean samples. Upon screening the indigenous Caribbean Q-M3 Y-chromosomes for additional variants seen in South American Indian populations (M19, M194, M199, SA01 [82,83]), none of these derived SNPs were observed.

The remaining Y-chromosomes from Trinidad and St. Vincent were of African (E1b1a) or likely European (I1, I2, R1a, R1b) origin. Overall, more than 80% of the Y-chromosome haplotypes in the indigenous Caribbean communities were non-indigenous to the Americas. The Vincentian samples were comprised of about half African and half European lineages, whereas the Trinidadian samples showed a larger frequency of paternal African (80%) than European (20%) lineages.

Analysis of Y-STR variation in the St. Vincent Garifuna and the FPC Trinidadians revealed a total of 30 distinct haplotypes in these populations, based on data from 17 loci (Table H in S1 File). Of these, 4 belonged to indigenous American haplogroup Q1a3a1a (Q-M3), 15 to African haplogroup E1b1a, and 11 to West Eurasian lineages (I1, I2, R1a and R1b). After being reduced to the SWGDAM locus set, these Y-STR sequences were used for all subsequent statistical and phylogenetic analyses.

Summary statistics for Q-M3 Y-STR haplotype data from both the comparative populations and the indigenous Caribbean groups were estimated to assess the paternal genetic affinities of the St. Vincent Garifuna and FPC Trinidadians. Due to their having a small number of indigenous Y-chromosomes, the data from Trinidadian and Vincentian communities were combined into a single “indigenous Caribbean” population. The gene diversity for the resulting indigenous Caribbean population was similar to that of the comparative populations (Tables 6 and 7). Similarly, the exact test for population differentiation showed the indigenous Caribbean population to be significantly different from only the comparative Brazilian samples (p = 0.018; Table I in S1 File). In addition, populations from regions geographically closest to the Lesser Antilles were not significantly different from the indigenous Caribbean population.

**Table 6:** Summary statistics for Y-STR haplotypes from Indigenous Caribbean populations belonging to haplogroups Q-M3.

	n	# Haplotypes	H (± SD)	Ave H (±SD)
St. Vincent &				
St. Vincent and Trinidad	4	4	1 (0.177)	0.517 (0.377)

Note: All haplotypes were defined using SWGDAM loci

**Table 7:** Summary statistics for Y-STR haplotypes (SWGDAM Loci) from comparative populations.

Geographic region		n	Number of Haplotypes	H (±SD)	Ave H(±SD)
Amazonian Basin	Brazil	23	11	0.893 (0.040)	0.442 (0.252)
Northern Tropics	Venezuela	8	8	1 (0.062)	0.646 (0.390)
	Colombia	19		1 (0.017)	0.585 (0.327)
	French Guiana	20	19	0.995 (0.018)	0.524 (0.295)
Central America	Honduras	25	23	0.993 (0.013)	0.547 (0.304)

Despite their general similarity, nearly all of the indigenous Caribbean Q-M3 haplotypes were unique to their respective populations, with only one haplotype being shared between a Vincentian Garifuna and a Kali’na individual from French Guiana [70]. The Kali’na speak a Cariban language and are believed to have arrived in the region extending French Guiana to Venezuela, the Guiana plateau, or a region near the Xingu River, a tributary of the Amazon River around 900 AD [70,84]. However, the genealogical history provided by the Vincentian participant did not indicate recent parentage from South America but instead a family history based in the Sandy Bay region of St. Vincent.

**Fig 6:**
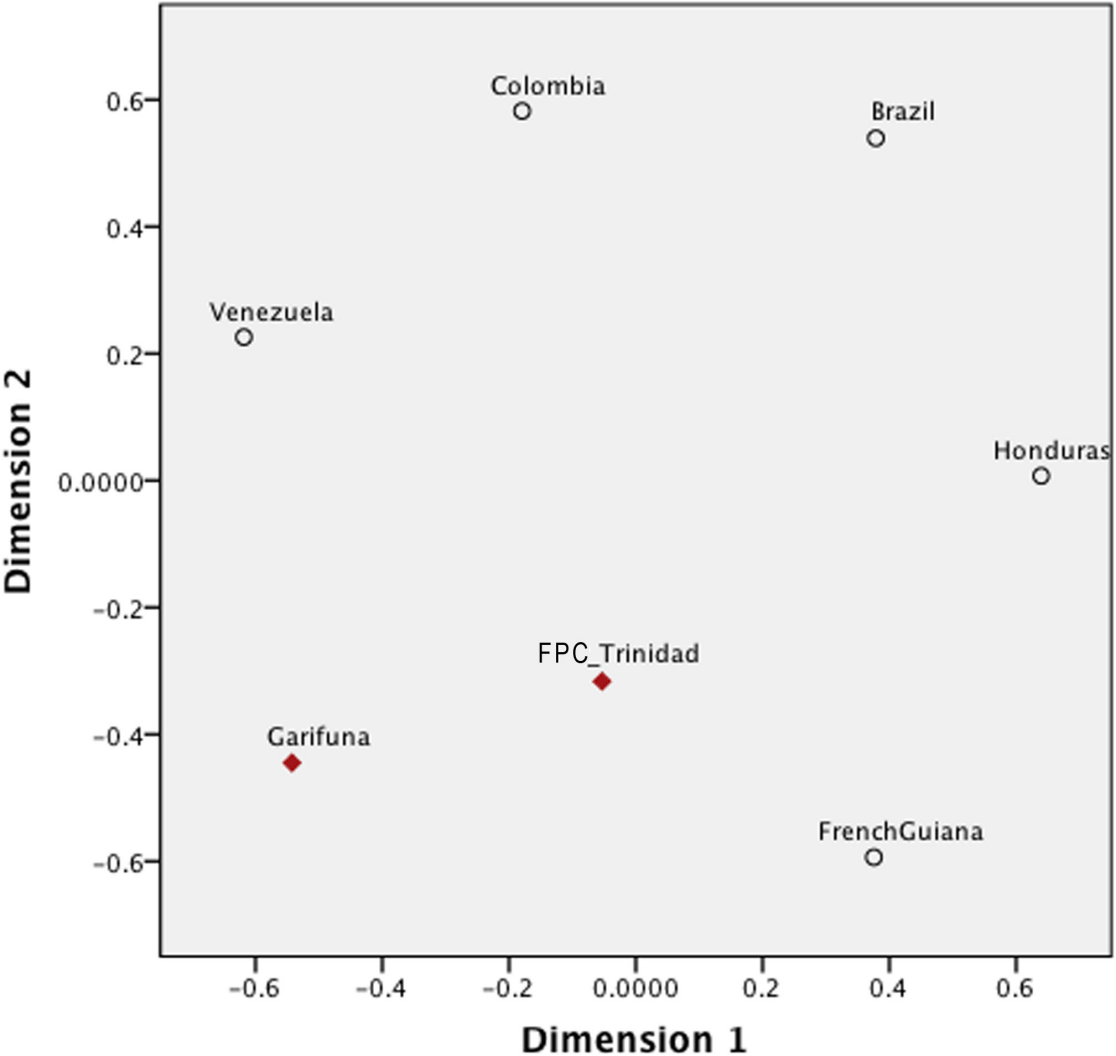
A MDS plot of RST estimates based on Y-STR haplotypes for Indigenous Caribbean and comparative Central and South American populations. The stress value of the plot is 8.7%.

R_ST_ values for the indigenous Caribbean and comparative populations were visualized through MDS (Table J in S1 File). In the resulting MDS plot, the Vincentian Garifuna and the FPC Trinidadians appeared closest to Venezuelans in Dimension 1 and French Guianans in Dimension 2 (Fig 6). The indigenous Caribbean groups were most distant from those in Colombia and Brazil, while also being somewhat distant from the Honduran group. Based on the p-values associated with the R_ST_ estimates (Table J in S1 File), the only significant difference occurred between Brazil and all other populations except Trinidad, which was represented by only a single haplotype in the MDS plot.

**Fig 7:**
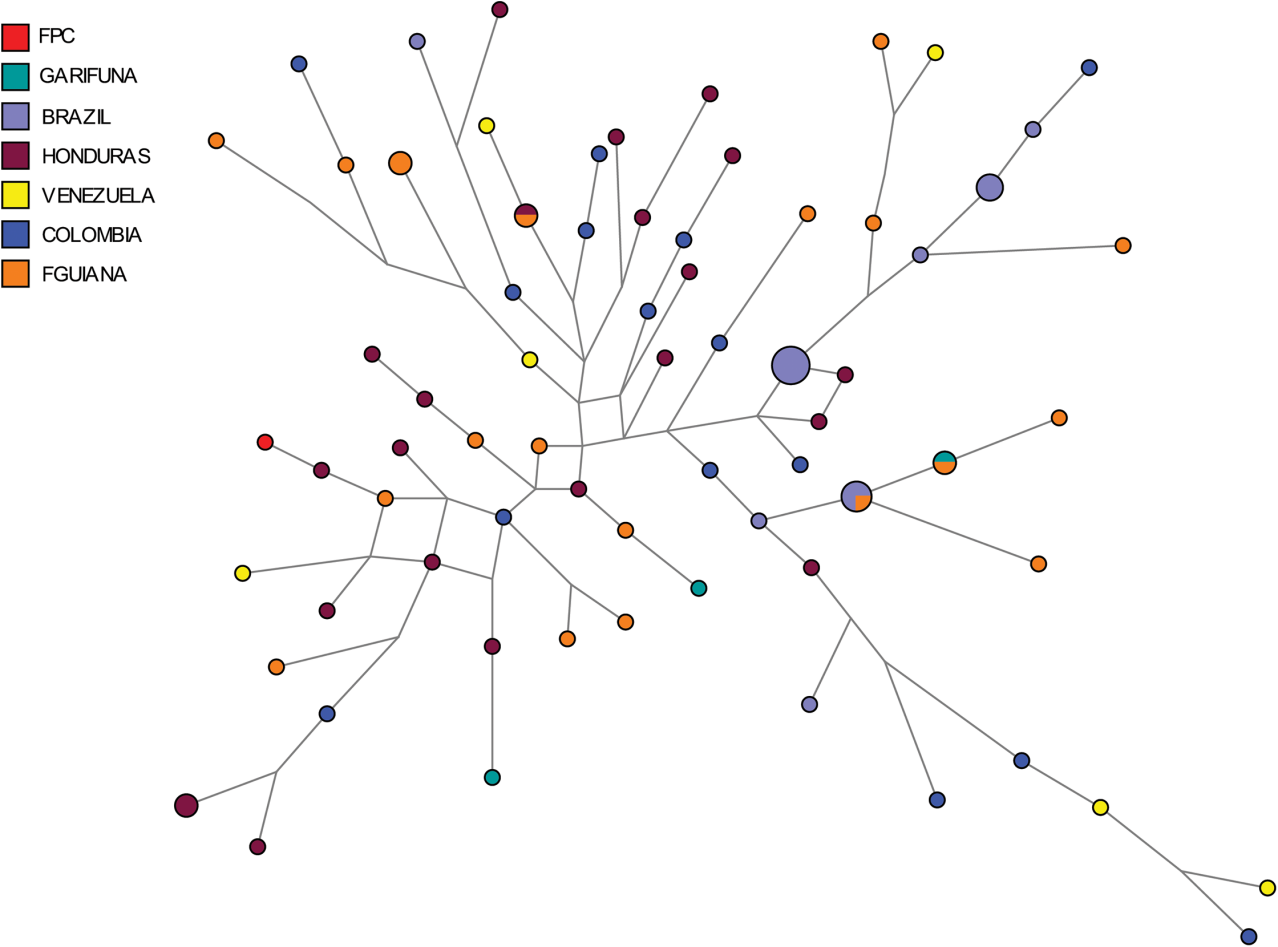
A median-joining network of Y-chromosome Q-M3 STR haplotypes from Indigenous Caribbean and comparative Central and South American populations. Populations represented in each node of the network are shown in different colors.

In the phylogenetic analysis of Q-M3 Y-STR haplotypes, those from indigenous Caribbean populations were distinct from each other, being located in different branches of the MJ network (Fig 7). With the exception of the abovementioned Vincentian Garifuna haplotype, no other indigenous Caribbean haplotypes were shared with comparative circum-Caribbean populations. The estimated coalescence date for the branches containing Vincentian and Trinidadian Caribbean haplotypes was 19,483 (± 2,420) ybp, a date more closely associated with the earliest peopling of the Americas. However, the cluster including only the haplotype shared between French Guiana and Vincentian Garifuna individuals dated to 2,841 (± 1,463) ybp, an estimate more closely matching the date for the peopling of the Lesser Antilles, based on archeological research.

## Discussion

### Maternal ancestries of indigenous Lesser Antillean communities

Analysis of mtDNA haplogroup diversity in the FPC Trinidadians and Vincentian Garifuna revealed relatively high frequencies of indigenous maternal lineages. Of the Native American haplogroups, only A2 and C1 were present in these communities. Broad surveys of genetic data from populations across the Caribbean indicate that haplogroup B2 and to a lesser extent, haplogroup D1, are rarely found in contemporary Caribbean populations, whereas A2 and C1 and their derivatives are more commonly observed [19,36,51,77,85–88]. This observation holds for ancient samples from the Lesser Antilles, as well. In a recent publication by Mendisco et al. (2015), haplogroup B2 was not observed in any of the thirteen samples from the Guadeloupe archipelago, and haplogroups A2 and C1 were equally represented, while D1 was present but not as common as the other haplogroups [89]. Thus, our results confirm a general regional pattern of indigenous mtDNA diversity in the Caribbean.

Interestingly, the FPC Trinidadians have a high frequency of A2 and low frequency of C1, whereas the Vincentian Garifuna show the opposite pattern. The majority of the Vincentian A2 and C1 mtDNAs appear to be founder haplotypes based on CR sequence data, suggesting limited diversity within this population. By contrast, within the Trinidadian FPC, only two individuals carried the A2 founder haplotype and none of the C1 founder haplotype. The fact that Vincentian Garifuna and FPC Trinidadians share no mtDNA haplotypes is noteworthy because of their relative geographic proximity. Similarly, estimates of heterozygosit**y** also indicate differences between the two indigenous Caribbean populations.

The frequencies of each mtDNA haplogroup may reflect the effects of genetic drift on patterns of genetic diversity on the islands, or perhaps reveal differences in the founding populations of the island groups as a result of multiple migrations into and throughout the region [3]. Despite the fact that archeological data suggest contacts between Greater Antillean and Mesoamerican populations, our data do not provide clear support for genetic relationships between indigenous groups from these regions. These results are consistent with previous genetic studies in the Caribbean, which reveal mtDNA differences between Greater and Lesser Antillean populations [52,86,87,90]. Overall, our data suggest a settlement process involving multiple migratory waves originating in the southern regions of the Caribbean in which the migrants replaced prior inhabitants and subsequently experienced relative isolation until the next expansion of human groups arrived from northern South America.

In addition to indigenous Caribbean ancestry, both of the study communities exhibited maternal lineages from Africa and, in the case of the Trinidadian FPC, South Asia. Most of the haplogroups observed in both communities were of African origin (L0, L1, L2, and L3). Haplogroup L2 was the most commonly observed African haplogroup within the Vincentians, while L2 and L3 were most common in the Trinidadians. Both of these lineages are frequently observed on other Caribbean islands, representing between 20–30% of the mtDNAs [51,77,87,91]. Previous genetic studies suggest that many African lineages found in the Americas derive from Niger-Kordofanian speaking populations in West and West-Central Africa, whose members arrived in the Americas primarily via the Trans-Atlantic slave trade [92–96]. In fact, Stefflova et al. (2011), found that, within Afro-Caribbean populations, 46% of their African ancestry could be traced back to the Guinea Bissau, Mali, Senegal and Sierra Leone regions, 29% to the region encompassing Niger, Nigeria, and Cameroon, and 25% to Angola [95].

These findings are generally concordant with the 19^th^ century census records of British colonial plantations in Trinidad. As illustrated by the 1813 census, the birthplaces of the majority of enslaved Africans in Trinidad were primarily from regions within the Bight of Biafa, Gold Coast, and the Bight of Benin [97]. Based on our initial archival work, the same kind of information was not recorded for enslaved populations in St. Vincent. However, since both St. Vincent and Trinidad were British colonies for much of their histories, the transplanted African peoples there might be expected to have similar origins.

#### St. Vincent mtDNA diversity

With regard to the Vincentian Garifuna, there are several noteworthy differences between this community and the Garifuna that were exiled to Honduras in the 18^th^ century. A previous analysis of Honduran Garifuna revealed only indigenous haplogroups A2 and C1 and several African haplogroups, but no European maternal lineages [77]. Although these findings are generally consistent with our results, the Vincentian Garifuna have a higher proportion (46%) of indigenous American mtDNAs than the Honduran Garifuna (16%). This difference may reflect the socio-political distinctions made by the British regarding the phenotypic appearance of Vincentians at the time of exile. In these distinctions, which ultimately separated families, those who were deemed light-skinned and presumed to descend from the island’s original inhabitants were called “Yellow (or Red) Caribs” and returned to St. Vincent, while those who were darker-skinned, presumably having both African and indigenous Caribbean parentage, were termed “Black Caribs” and exiled to Central America [20]. Furthermore, in their analysis of ancient samples from the Guadeloupe Archipelago, Mendisco and colleagues (2015) found two mitochondrial haplotypes that matched those in Honduran Garifuna individuals. They suggested that the shared haplotypes were the result of the historical connection between the Vincentian Garifuna and those that were exiled to Central America [89]. In our current study, all of the haplogroup C1 haplotypes (haplotypes 17–22 in Table **B** in S1 File) observed in St. Vincent matched ancient samples from two individuals in Marie Galante and La Désirade, respectively. The matching ancient samples come from the post-Saladoid period and date to AD 1289–1445, suggesting continuity of these particular genetic lineages within Lesser Antillean populations.

#### Trinidadian mtDNA diversity

In addition to indigenous American and African mtDNA haplogroups, two members of the Trinidadian indigenous community also had maternal lineages of South Asian origin that belonged to haplogroup M33a [75]. This finding was not entirely surprising, given the colonial history of Trinidad. Shortly after Emancipation in 1838, formerly enslaved African Trinidadians moved en mass away from plantations, creating a labor void that was filled by South Asian indentured laborers [98,99]. Nearly 142,000 laborers arrived in Trinidad between 1845–1917 during the height of South Asian indenture, with many coming from provinces in the northwest, northeast (Bengal and Bihar), Awadh (also called Oudh), and Punjab regions [98,100]. Although haplogroup M lineages are ubiquitous in India [101], M33a is most commonly found among tribes in western India [102,103], while also occurring at high frequency (~55%) among the Garo in the Bengal region of northeast India [75]. Considering both historic and genetic data, our results therefore suggest that some members of the Trinidad FPC have maternal genetic ancestry tracing back to the northern regions of India.

#### mtDNA diversity in the circum-Caribbean region

Analysis of mtDNA variation of all of the haplotypes observed in the indigenous Caribbean populations illustrates that the indigenous Caribbean groups have similar levels of genetic diversity relative to the comparative populations. However, when African and South Asian haplotypes are removed, mtDNA diversity in the indigenous Caribbean groups decreases considerably. This reduction in mtDNA diversity in indigenous Caribbean populations may be attributed to several factors including the effects of genetic drift, the loss of indigenous lineages in historical times, and possibly endogamy which may have led to the enrichment of certain mtDNA haplotypes in the populations. Furthermore, patterns of genetic diversity differed between the Vincentian Garifuna and the FPC Trinidadians. This difference suggests that both groups possess specific sets of mtDNA haplotypes that are reflective of their unique histories, including the possibility of having different ancestral origins in South America.

In the MDS plot containing indigenous Caribbean and comparative circum-Caribbean mtDNA data (Fig 2), both study populations are positioned close to South and Central American populations, suggesting some genetic similarities to them. However, given the effects of genetic drift within Caribbean groups, as indicated by their low mtDNA diversity and high between-population variances, it may be difficult to clearly differentiate population affinities for them. Nonetheless, linguistically related groups that are geographically proximate cluster together, e.g., Tupian and Jêan speakers, whereas the indigenous Caribbean groups do not align exclusively with populations from a specific language family. This result is not entirely surprising, as previous studies examining the relationship between linguistic affiliations and genetic markers generally indicate a weak relationship between language and genetic diversity for Native American populations, although this relationship becomes stronger at more local levels [104–106]. However, the linguistic data indicate that indigenous Caribbean populations spoke languages belonging to both Maipurian (Arawakan) and Cariban languages families [2]. Thus, additional genetic data from indigenous populations belonging to both of these language families will be needed to resolve the relationship between the genetic background and linguistic affiliation of indigenous Caribbean populations.

Analysis of mtDNA diversity in the indigenous Caribbean and Anglophone Caribbean populations shows that indigenous Caribbean groups are genetically distinct along the maternal line from the greater populace. However, the Vincentian Garifuna show greater affinities with the other island populations than the Dominicans and Trinidadians. This pattern may reflect the dual African and indigenous Caribbean ancestry of the Vincentian Garifuna and their subsequent cultural and genetic exchanges with indigenous Caribbean peoples as documented by historic sources [20,77,107]. The distinctiveness of the Dominican population may also reflect differences in the indigenous and colonial population history of Dominica [108].

Both indigenous Caribbean communities had founder A2 and C1 haplotypes and these were also observed in Puerto Rican populations [52,90]. Yet, the derived Vincentian haplotypes were largely distinct from those of both Trinidadians and Puerto Ricans. This observation suggests that, while there may be some deeper shared ancestry between indigenous Caribbean communities, each has become genetically differentiated from the others through genetic drift, separate migration events or stochastic lineage loss.

Coalescence analysis of the Caribbean haplotypes indicates that haplogroup C1 is younger (2861 ybp) than haplogroup A2 (8,489 ybp) in the Lesser Antilles. These dates are generally congruent with archeological data from the Caribbean, which indicate the Lesser Antilles were initially inhabited as early as 7,200 ybp in a series of migrations from circum-Caribbean regions [2,3]. However, given certain caveats about mutation rates of mitochondrial loci [109,110], the limited number of haplotypes used in the coalescence estimate, and the reduced diversity consistent with founder effects, these dates should be viewed as approximations. On the other hand, we have previously noted differences in the ages of mtDNA haplogroups in our study of Puerto Ricans, and these may possibly reflect multiple migrations into the Caribbean Basin [52]. It is, therefore, plausible that a similar scenario explains our data from the Lesser Antilles.

### Paternal ancestries of indigenous Lesser Antillean communities

#### Indigenous paternal ancestry

Based on the summary statistics, indigenous Caribbean populations show similar levels of paternal genetic diversity to comparative Caribbean populations, although having distinct sets of Y-STR haplotypes. However, as seen in Fig 7, indigenous Caribbean and most of the comparative Central and South American populations are not clearly distinctive from one another. This result may be attributable to our using a limited set of Y-STRs in the analysis of population affinities that provide a relatively low resolution of haplotypic diversity.

In addition, the Vincentian and Trinidadian populations lack Q-M3 Y-chromosomes bearing derived SNPs (M19, M194, M199, SA01) seen in related haplotypes in indigenous populations from western South America [83,111]. The absence of these derived SNPs suggests stronger genetic affinities of indigenous Caribbean groups with indigenous Central and South American populations in geographic areas thought to represent potential source areas for demographic expansions into the Caribbean (i.e., Brazil and Venezuela) as opposed to western or central South America. The NRY data may further reflect the effects of genetic drift on the indigenous Caribbean communities, or possibly indicate that these communities are reservoirs of genetic diversity reflective of ancient settlement and migrations in the Caribbean.

Moreover, our data provide the first evidence of indigenous Y-chromosomes in Caribbean populations from the Lesser Antilles. Recently, Marcheco-Teruel et al. (2015) [112] identified indigenous Y-chromosomes in Cuba, an island in the Greater Antilles. Prior to these studies, it appeared that indigenous American paternal haplogroups had been entirely replaced by those from colonizing populations as a result of European conquest and colonization [113–116]. Our data firmly demonstrate that indigenous American haplotypes are present in the Lesser Antilles today despite these disruptive historical events.

Phylogenetic analysis shows that Q-M3 STR haplotypes in the FPC Trinidadian and Vincentian Garifuna differ from each other and have distinct genetic affinities with those from different areas of Central and South America (Honduras, Brazil, Venezuela, and Colombia). This result was intriguing because of the small geographic distance between the two indigenous Caribbean groups and the circum-Caribbean populations, and the history of pre-Colombian migration within the region [86,117]. Thus, despite their small number, the fact that most Caribbean indigenous Y-chromosomes represent unique derived Q-M3 haplotypes suggests they either evolved through genetic drift and isolation on each island or arrived through separate population expansions from South America.

Indigenous Caribbean peoples are thought to be culturally related to Arawakan speakers of mainland South America, partly due to their having incorporated loan words from Cariban speaking groups [9,17]. Our genetic data point to connections between the Cariban speaking Kali’na and indigenous Caribbean populations. In addition to French Guiana, Kali’na people are found in present day Venezuela, British Guyana, Suriname, and Brazil [84]. The demographic distribution of Kali’na on the South American mainland may also help to explain the placement of Lesser Antillean Q-M3 haplotypes within the MJ network. Even so, the relationship between the Kali’na and Vincentians is intriguing, and calls into question the conventional assertions regarding the relationship between indigenous Caribbean and mainland South American populations.

#### Non-native paternal ancestry

Y-chromosome variation in indigenous Caribbean groups also shows evidence of significant historical gene flow from African and European males. African haplotypes, represented by haplogroup E1b1a, likely entered the indigenous Caribbean communities as a result of the Trans-Atlantic trade in enslaved Africans [118,119]. E1b1a occurs at the highest frequencies in sub-Saharan African (80%) and West-Central African populations (60%) [120]. According to 19^th^ century British plantation censuses [97], most of the enslaved Africans in the southern British Caribbean colonies came from people in the Bight of Biafra. Accordingly, paternal ancestry from Africans in the west and west-central regions of the continent is expected and is consistent with historical records [121].

Several predominately European haplogroups were also observed in the indigenous Caribbean communities. Like their African counterparts, European genetic contributions to indigenous Caribbean communities initially began during the colonial period [122–124]. Haplogroup R1 is frequently found among Europeans, being observed in greater than 50% of European men [125], although different sub-lineages are found throughout South, Central, and West Asia, as well as the Sahel region of Africa [125,126]. The most common R1 sublineages, R1a and R1b, are both observed in the indigenous Caribbean populations. R1a is distributed from southern Siberia to central regions of Eastern Europe and South Asia [127], whereas R1b is found primarily among Western European peoples, and to a lesser extent, throughout Asia [125,128]. R1b haplotypes have also been found, although at much lower frequencies, in certain African populations from central-western Africa [126,129]. Given the frequency of haplogroups R1a and R1b within the populations that colonized the Caribbean, most notably Western European groups, the presence of these paternal lineages in the Caribbean are clearly suggestive of their introduction during European colonization, and thus gene flow from the colonial power into native populations. However, further SNP genotyping will be needed to clearly distinguish African R1b from European R1b haplotypes.

Two other non-native paternal lineages observed in the indigenous Caribbean communities were haplogroups I1 and I2. These haplogroups are ubiquitous in Europe, accounting for nearly 18% of European Y chromosomes [130]. Haplogroup I1 is most commonly found among Scandinavian populations and also appearing at appreciable frequencies in Ireland, England and Scotland [130,131], while haplogroup I2 is most frequently found among Slavic groups in Eastern Europe as well as in Europeans near the Mediterranean and Atlantic coasts [130]. Thus, the presence of these haplogroups in Vincentians and Trinidadians is also indicative of gene flow from European peoples.

Also noteworthy is the pattern of European paternal ancestry among the indigenous Caribbean communities. In the Vincentian Garifuna, nearly half of the Y-chromosomes were of European ancestry while, in the Trinidadian FPC, less than one-quarter of the Y-chromosomes were. This observation could reflect differences in the histories of the two indigenous communities. When comparing the levels of European paternal ancestry in other Caribbean communities with a strong indigenous Caribbean influence, such as those in Dominica and St. Lucia [132], indigenous Vincentians appear most comparable to these island populations. Previous work suggests that, like the Vincentian Garifuna, groups from both Dominica and St. Lucia have nearly half African and half European paternal ancestry [51]. Thus, the FPC Trinidadians appear to diverge from the general pattern of roughly equal contributions of African and European males to the paternal genetic make-up of Caribbean populations with known indigenous ancestry. Given that Spanish surnames have historically been markers of indigenous ancestry within Trinidad [21], additional genetic and ethnographic data are needed to further clarify the pattern of European paternal ancestry in the FPC community.

## Conclusions

Conventionally, archeological and linguistic data have been the primary sources of information used to infer details concerning the peopling of the Caribbean Basin and these lines of evidence remain important for contextualizing the genetic record. As indicated by archeological and linguistic data, the diversity in the Caribbean reflects both migration and contact, and also isolation and differentiation, over thousands of years. Based on these data, scholars have hypothesized that Vincentian and Trinidadian indigenous groups would more strongly resemble indigenous groups from northern South American because the Carib/Arawakan expansion took place recently, whereas the Taíno groups in the Greater Antilles would resemble other South American groups, and perhaps also Mesoamerican populations, due to their representing an earlier genetic stratum of human populations involved in the initial peopling process. Genetic data from the Vincentian Garifuna and Trinidad FPC, though limited by sample size, support the South American origin and possible Mesoamerican connections of indigenous Caribbean peoples. Furthermore, our genetic data provide more information about the impact of colonization on indigenous Caribbean peoples. While African and European paternal lineages replaced many of those from indigenous Caribbean groups, there remains evidence of indigenous paternal ancestry in contemporary indigenous Caribbean communities.

The current study provides a glimpse of the genetic history of the region’s first peoples. Future work will include more comprehensive analyses of mitogenome sequences and Y-chromosome diversity in indigenous Caribbean groups. In addition to the uni-parental genetic loci, analyses of autosomal variation in these populations will further illuminate genetic history of the region.

## Genetic Tools

Arlequin: http://cmpg.unibe.ch/software/arlequin35/

DnaSP: http://www.ub.edu/dnasp/

Haplogrep: http://haplogrep.uibk.ac.at/

Haplogroup Predictor: http://www.hprg.com/hapest5/

Network: http://www.fluxus-engineering.com/sharenet.htm

## Supporting Information

S1 File Table A. mtDNA SNP haplotypes in St. Vincent and Trinidad Table B. mtDNA control region sequences in St. Vincent & Trinidad Table C. mtDNA HVS1 Sequences in St. Vincent & Trinidad Table D. GenBank Accession numbers for mtDNA HVSI sequences Table E. Exact tests of based on mtDNA HVS1 sequence dat Table F. *F*_ST_ estimates based on mtDNA HVS1 sequence data Table G. Y-chromosome SNP haplotypes in St. Vincent & Trinidad Table H. Y-chromosome STR haplotypes in St. Vincent & Trinidad Table I. Exact tests of based on Y-chromosome STR haplotype data Table J. R_ST_ estimates based on Y-chromosome STR haplotype data (XLSX)
